# Tributyrin, a Butyrate Pro-Drug, Primes Satellite Cells for Differentiation by Altering the Epigenetic Landscape

**DOI:** 10.3390/cells10123475

**Published:** 2021-12-09

**Authors:** Robert L. Murray, Wei Zhang, Jianan Liu, Jason Cooper, Alex Mitchell, Maria Buman, Jiuzhou Song, Chad H. Stahl

**Affiliations:** 1 Department of Military and Emergency Medicine, F. Edward Hebert School of Medicine, Uniformed Services University, Bethesda, MD 20814, USA; robert.murray@usuhs.edu; 2 Department of Animal and Avian Sciences, University of Maryland, College Park, MD 20742, USA; lacee689@gmail.com (W.Z.); emilyliu@terpmail.umd.edu (J.L.); rpaulo@umd.edu (J.C.); chadstahlphd@gmail.com (A.M.); ecooper7@umd.edu (M.B.); songj88@umd.edu (J.S.)

**Keywords:** satellite cell, butyrate, epigenetics, ChIP-Seq, myogenic differentiation, HDAC inhibitor

## Abstract

Satellite cells (SC) are a population of muscle resident stem cells that are responsible for postnatal muscle growth and repair. With investigation into the genomic regulation of SC fate, the role of the epigenome in governing SC myogenesis is becoming clearer. Histone deacetylase (HDAC) inhibitors have been demonstrated to be effective at enhancing the myogenic program of SC, but their role in altering the epigenetic landscape of SC remains undetermined. Our objective was to determine how an HDAC inhibitor, butyrate, promotes myogenic differentiation. SC from tributyrin treated neonatal piglets showed a decrease relative to SC from control animals in the expression of enhance of zeste homologue-2 (EZH2), a chromatin modifier, ex vivo. Chromatin Immunoprecipitation-Sequencing (ChIP-Seq) analysis of SC isolated from tributyrin treated pigs showed a global reduction of the tri-methylation of lysine 27 of histone H3 (H3K27me3) repressive chromatin mark. To determine if reductions in EZH2 was the primary mechanism through which butyrate affects SC behavior, SC were transfected with siRNA targeting EZH2, treated with 0.5 mM butyrate, or both. Treatment with butyrate reduced paired-box-7 (Pax7) and myogenic differentiation-1 (MyoD) gene expression, while siRNA caused reductions in EZH2 had no effect on their expression. EZH2 depletion did result in an increase in differentiating SC, but not in myotube hypertrophy. These results indicate that while EZH2 reduction may force myogenic differentiation, butyrate may operate through a parallel mechanism to enhance the myogenic program.

## 1. Introduction

Tissue-specific stem cells have the potential to self-renew or differentiate into mature cells, contributing to tissue growth and homeostasis. Muscle specific stem cells, satellite cells, maintain the ability to self-renew or differentiate and are required for post-natal muscle growth and repair [1,2,3]. The use of novel gene sequencing technology in the investigation of the genomic regulation of satellite cell fate has provided insight into how the satellite cell epigenome governs myogenesis [4,5]. Specifically, the changes in the chromatin structure and the regulators that direct these changes modulate satellite cell activation, proliferation, and differentiation [6,7,8,9]. Histone deacetylase (HDAC) inhibitors have proven to be effective at enhancing the myogenic program of satellite cells [10,11,12], but their precise mode of action in remodeling the epigenetic landscape of satellite cells remains undetermined. One such HDAC inhibitor, butyrate, is a short-chain fatty acid that has been validated as an effective promoter of myogenic differentiation [10], and while the mechanism remains uncertain, indications allude to a modification of the epigenetic status [13]. We recently demonstrated that enteral supplementation of the butyrate prodrug tributyrin in a neonatal growth model resulted in altered satellite cell behavior and increased muscle growth [14]. Determining the degree to which these presumptive alterations to the satellite cell epigenome are triggered by tributyrin supplementation would be of great significance in combating muscle disease and improving muscle growth.

Satellite cells lie along the muscle fiber beneath basal lamina beside plasma membrane [15] and remain in a “poised” state, ready to activate, proliferate, and differentiate upon molecular and mechanical cues [8,16,17]. The epigenetic status of satellite cells has been well defined through their myogenic lineage [5,18], marked by their timely expression of a hierarchal network of transcription factors [2,19]. The paired-box 7 (Pax7) transcription factor is a hallmark of satellite cell identification and is required for postnatal muscle growth and repair [20,21]. Upon satellite cell activation, Pax7 is upregulated and cells will proliferate and begin expressing the basic helix-loop-helix myogenic regulatory factor (MRF), myogenic differenation-1 (MyoD) [22]. The transcription factor MyoD is a master regulator of satellite cell lineage fate and is responsible for the expression of muscle specific genes through its initiation of the differentiation program [23]. Satellite cells that do not express MyoD or have high levels of Pax7 expression compared to MyoD, will self-renew and go back to a quiescent state, reserved for the next round of stimulation and activation [24,25]. MyoD has also been found to play a role in repressing the muscle differentiation genes that it is associated with until differentiation cues are present [26], and not all the genes that MyoD associates with are expressed at the same time [27]. These findings describe the integral arrangement and temporal spacing of gene expression during the myogenic program. Another MRF, myogenin is also required for proper satellite cell terminal differentiation [28] and is regulated by MyoD. In both a direct and indirect manner, MyoD recruits chromatin remodeling enzymes to the myogenin promoter, allowing for the binding of MyoD to the promoter region and allowing for transcription [29,30]. Myogenin target genes include those responsible for appropriate satellite cell fusion and contractile protein formation [31,32]. Myogenin expression marks the transition from myoblast proliferation to myocyte differentiation [33] and the down-regulation of Pax7, as the two are mutually exclusive [34]. Upon pro-myogenic differentiation cues, regulatory remodeling of chromatin around these transcription factors has been shown to govern the rate at which satellite cells progress through their myogenic program [4,5].

The subset of myogenic progenitor cells that make up the satellite cell pool after embryogenesis lie in quiescence and rely on a highly coordinated transcriptional network that govern maintenance, activation, and differentiation. Satellite cell commitment to its myogenic lineage is regulated and defined by the epigenome [35], and with new chromatin immunoprecipitation sequencing (ChIP-Seq) technology, these corresponding epigenetic signatures have been well defined [36]. Satellite cell quiescence is maintained by the absence of the repressive trimethylated lysine 27 residue on the histone H3 protein (H3K27me3) and the presence of H3K4me3 [36]. Upon satellite cell activation, most of the H3K4me3 marks remain, but repressive H3K27me3 marks are deposited on muscle specific genes (MyoD, myogenin; MyoG) by the Polycomb group (PcG) proteins in association with the transcriptional repressor HDAC1 [9]. Satellite cells will then continue to either self-renew, or asymmetrically divide and give rise to myogenic daughter cells that begin to express MyoD [3]. These committed daughter cells (termed myoblasts) retain the permissive H3K4me3 but lose the bivalent H3K27me3 mark, allowing for increased MyoD expression [9]. Prior to myoblast differentiation, MyoD associates with HDAC1 resulting in silencing of MyoD-dependent transcription of muscle specific genes [37,38]. Myoblasts continue to proliferate until pro-differentiation cues lead to a slowing of Pax7 transcription through the removal of H3K4me3 and deposition of the repressive H3K27me3 marks by the Polycomb repressive complex 2 (PRC2) [7]. On specific activation cues, recruitment of acetylated lysine residues (H3K27ac) [39] followed by concomitant removal of the H3K27me3 marks around the myogenin promoter to allow for transcriptional activation and terminal differentiation to begin [40]. This removal is consistent with a loss of the catalytic subunit, EZH2, of the PRC2 protein which regulates the methyltransferase capability of PRC2 and is strongly correlated with the mediation of satellite cell differentiation [41]. The HDAC inhibitor butyrate has been shown to disrupt and downregulate EZH2 expression, resulting in a reduction of H3K27me3 marks around lineage specific genes [13].

The use of HDAC inhibitors in satellite cell cultures and animal growth studies have yielded positive results with regards to muscle differentiation [10] and growth [42,43,44], respectively. Also, EZH2 depleted satellite cells have shown an increased expression of myogenin, but the timing and effect on the differentiation program have not been specifically defined [40]. While the differing effects of using butyrate on in vitro myogenesis are clear, in vivo studies are lacking. We demonstrated that feeding tributyrin resulted in altered satellite cell activity with an increase in muscle growth [14]. Further refinement of the differences within this satellite cell population is warranted due to differences in both in vitro and in vivo behavior. Determining the molecular regulators affected by tributyrin supplementation with regards to muscle growth may provide targets for manipulation in treating myopathies and promoting muscle growth. Additionally, we attempted to determine if butyrate’s effect on satellite cell myogenesis is mediated entirely through EZH2.

## 2. Materials and Methods

To assess the impact of dietary tributyrin inclusion on in vivo satellite cell epigenetics, satellite cells isolated from a previous study were used for our experiments (see [14] for detailed animal study protocol). Tributyrin inclusion was on a dry matter basis and coconut oil was used to ensure the diets were isoenergetic. Body weight and feed intake were recorded daily for the duration of the 21d feeding trial. At sacrifice, *Longissumus dorsi* (LD) muscle was collected and used for all tissue analysis and satellite cell isolation.

Satellite cells were isolated according to a procedure modified from Doumit and Merkel [45] and Allen et al. [46]. Briefly, LD muscle was excised from neonatal piglets, and thoroughly minced with scissors. The resulting tissue fragments were subjected to protease digestion (1.25 mg/mL protease from Streptomyces griseous (Pronase; Sigma-Aldrich, St. Louis, MO, USA) for 1 h at 37 °C). Satellite cells were then separated from tissue fragments by differential centrifugation. Cells were then pre-plated on uncoated 15 cm tissue culture dishes for 2 h (37 °C, 5% CO_2_) in proliferative growth media (PGM, DMEM + 10% FBS + antibiotics—100 U/mL penicillin, 100 µg/mL streptomycin, 10 µg/mL gentamycin; Thermo Fisher Scientific, Waltham, MA, USA) and then placed on tissue cultured treated dishes coated with Poly-L-lysine (100 µg/mL ddH_2_O, Sigma-Aldrich, St. Louis, MO, USA) and fibronectin (10 µg/mL PBS, Sigma-Aldrich) in PGM until they reached 50% confluence (37 °C, 5% CO_2_). Isolated cells were then released with 0.05% Trypsin (Thermo Fisher Scientific, Waltham, MA, USA) and either plated for our studies or used for ChIP-Seq analysis. Cell preparations that were identified as >97% Pax7^+^ were used for all downstream analytics [47].

The effect of dietary tributyrin inclusion on ex vivo satellite cell expression of EZH2 was analyzed through their myogenic progression under proliferative and differentiative conditions. Satellite cells were seeded at 2500 cells/cm^2^ in PGM on to plates coated with Poly-L-lysine and fibronectin. After a 24 h attachment period, satellite cell proliferated for 48 h and were then induced to differentiate (DM, DMEM + 2% horse serum; Sigma-Aldrich, + antibiotics) for an additional 48 h with complete media changes daily. Total RNA was isolated (RNeasy; Qiagen, Hilden, Germany) at each 24 h time point for gene expression analysis.

The effect of butyrate and the role of EZH2 in the myogenic progression of satellite cells was determined in satellite cells, isolated from pigs not fed tributyrin. Satellite cells were plated and allowed to proliferate in PGM until around 80% of confluence, were transfected, treated with 0.5 mM sodium butyrate (NaBu), or both (Mixed) for 24 h and then induced to differentiate. Cells were transfected with 100 nM duplexed siRNA targeting the EZH2 transcript (siEZH2) (Dharamcon, Lafayette, CO, USA) or a scrambled control (siScrambled) (MISSION Universal Negative Control; Sigma-Aldrich, St. Louis, MO, USA) using Lipofectamine 2000 (Thermo Fisher Scientific, Waltham, MA, USA) for 24 h according to the manufacturer’s protocol. Transfection efficiency was around 80% with around 70% knockdown efficiency in EZH2 transcript. Total RNA was collected at the time of treatment (D-24) and every 24 h for 96 h (72 h after differentiation, D+72). At the D+72 h time point, satellite cells were fixed and immunostained for myosin heavy chain expression as an indicator of terminal differentiation and myotube formation.

Total RNA was isolated from SC using tri-reagent (Thermo Fisher Scientific, Waltham, MA, USA) and purified with RNeasy spin columns (Qiagen) according to the manufacturer’s protocols. Total RNA was then quantified using the Quant-iT RiboGreen assay (Thermo Fisher Scientific, Waltham, MA, USA) according to the manufacturer’s protocol. The RNA was used to generate cDNA using the SuperScript IV First-Strand Synthesis System (Thermo Fisher Scientific, Waltham, MA, USA). These samples were then treated with the RNase H to ensure the removal of RNA. The resulting cDNA was quantified with the Quant-iT OligoGreen assay (Thermo Fisher Scientific, Waltham, MA, USA). Total RNA and cDNA quantification were performed on the Synergy HTX microplate reader using the Gen 5.0 v3.0 software (BioTek Instruments, Winooski, VT, USA). cDNA was used for qRT-PCR using Bio-Rad’s (Hercules, CA, USA) CFX96 Touch Real-Time PCR Detection System and iQ Multiplex Powermix or iQ SYBR Green Supermix. Analysis of gene expression and amplification plots were executed with the CFX Manager Software (version 3.1, Bio-Rad, CA, USA). Primers and probes for the multiplexed myogenic genes of interest and EZH2 expression (singleplex) were normalized to RPL4 using ^∆∆^CT method (Table S1). For each assay, samples were amplified for 45 s at 60 °C for 40 cycles. Primers and probes were designed by Integrated DNA Technologies (Coralville, IA, USA).

Satellite cell cultures used for immunocytochemical analysis were plated onto Matrigel coated plates (1:10 dilution; Corning, Corning, NY, USA) to determine terminal differentiation and reveal myotube formation and satellite cell fusion. Satellite cells were analyzed for the expression of the contractile protein myosin heavy chain (MyHC) after 72 h of differentiation. Satellite cells were fixed in 4% paraformaldehyde and permeablized in 0.1% Triton X-100 (Sigma-Aldrich, St. Louis, MO, USA) in PBS. Fixed cells were then blocked with 10% goat serum in PBST (0.1% Tween-20 in PBS) for 1 h at room temperature. Following blocking, samples were incubated overnight at 4 °C with primary mouse monoclonal anti-MyHC at 10 µg/mL (Roche, Indianapolis, IN, USA). Primary antibodies were removed and then incubated with the secondary antibody (AlexaFluor 488 goat anti-mouse IgG at 1:500 dilution; Jackson Immunoresearch, West Grove, PA, USA) in 5% goat serum for 1 h at room temperature. Nuclei were visualized with 4′,6-diamidino-2-phenylindole (DAPI) (Sigma-Aldrich, St. Louis, MO, USA). Images were visualized on a Zeiss AxioObserver Z.1 and analyzed with ZenPro automated image analysis suite (Carl Zeiss AG, Oberkochen, Germany); >400 cells/animal were assessed for myosin heavy chain expression from at least eight fields of view.

ChIP libraries were constructed from primary satellite cells (*n* = 3/treatment group) and were utilized to perform ChIP-Seq analysis as previously described [48]. Briefly, approximately one million cells/sample were used to obtain formaldehyde-crosslinked chromatin that was fragmented into smaller sizes (around 150–300 base pairs) by sonication (Cell Disrupter 350; Heat Systems Ultrasonics, Plainview, NY, USA) and micrococcal nuclease (New England Biolabs, Ipswich, MA, USA) digestion. Chromatin was then immunoprecipitated with ChIP-grade H3K27me3 (ab6002) and H3K27ac (ab4729) antibodies (Abcam, Cambridge, UK) and purified to obtain immunoprecipitated DNA. ChIP DNA was repaired by using NEBNext End repair module (New England Biolabs, Ipswitch, MA, USA) and 3′A addition was performed using NEB Klenow Fragment (New England Biolabs). Then, adaptors (Illumina Inc, San Diego, CA, USA) were ligated to the repaired end with T4 DNA ligase (New England Biolabs, Ipswitch, MA, USA). After PCR amplification, DNA fragments of 200–500 bp were selected from the agarose gel. The resulting library was sequenced on the Illumina HiSeq 2000 single-end system (Illumina Inc, CA, USA).

Raw sequence data quality was checked by FastQC [49]. Bowtie, an ultrafast memory-efficient short read aligner was used to align reads to the pig reference genome Sscrofa10.2 (Swine Genome Sequencing Consortium) [50]. No trimming process was used because of high data quality. After quality control and alignment, peak-calling was achieved by using Model Based Analysis of ChIP-Seq (MACS) [51]. Identification of differentially enriched regions between treatment and control groups were accomplished by DiffBind package in R (version 3.2.3, Buffalo, NY, USA) [52]. Peaks identified with MACS (BAM) were used as input data for DiffBind. EdgeR analysis was run in the DiffBind package and used for Trimmed Mean of M-values normalization. The significance cut-off value for identification of differential regions was set to <0.1 false discovery rate (FDR) for H3K27ac and <0.05 FDR for H3K27me3. Next, the ChIPpeakAnno package in R was used to annotate genomic features of those identified differential enrichment regions [53]. ChIPpeakAnno allows the extraction of information overlaps, distances, and relative positions of requested genetic features. Gene ontology (GO) enrichment analysis was performed on the online DAVID Bioinformatics Resources 6.8 to highlight the most relevant GO terms from the list of related gene annotated by ChIPpeakAnno [54].

All other data were statistically analyzed using an F-test in ANOVA (GraphPad Prism 7, GraphPad Software, Inc., La Jolla, CA, USA). In the case of a significant F-test, multiple mean comparisons were analyzed using a Tukey’s adjustment. A student’s t-test was used to analyze treatment mean differences where indicated. Data normality was checked with D’Agostino-Pearson omnibus test. A probability of *p* ≤ 0.05 was considered significant and a *p*-value between 0.05 and 0.10 (0.05 < *p* ≤ 0.10) was considered a trend.

## 3. Results

### 3.1. Tributyrin’s Effect on EZH2 Gene Expression in Satellite Cells

Muscle tissue and isolated satellite cells were assessed for tributyrin’s impact on EZH2 gene expression. We previously had found that myogenin expression was upregulated after satellite cells had been induced to differentiate; however, this did not correlate with a specific downregulation of Pax7 under proliferative conditions [14]. While EZH2 and the PRC2 complex is essential at maintaining the pool of muscle progenitor cells, it is not required for the terminal differentiation processes [41]. The downregulation of EZH2 is linked to the start of the myogenic program, but EZH2 protein degradation precedes transcriptional repression [55]. The reduction in EZH2 gene expression indicates that this may be one of the final steps of myoblast differentiation.

**Figure 1:**
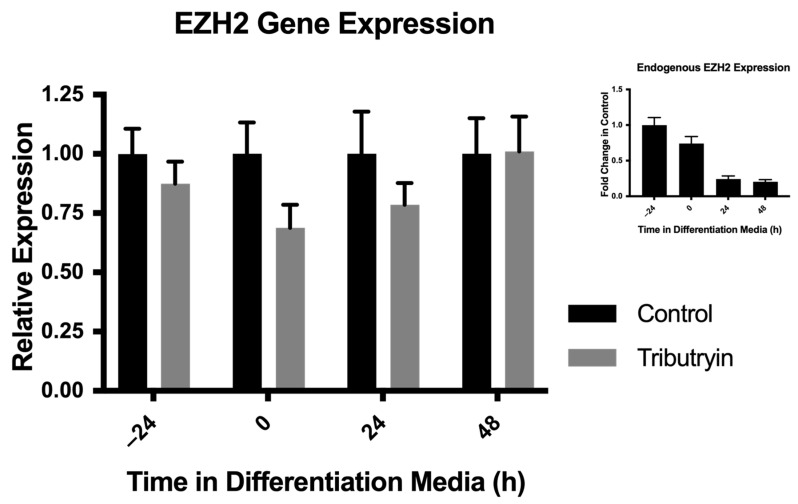
Enhancer of zeste homologue-2 (EZH2) expression ex vivo from primary satellite cells. Satellite cells were isolated from neonatal piglets fed a control milk replacer or a milk replacer supplemented with 0.5% Tributyrin. Satellite cells were cultured under proliferative conditions until around 90% confluence and then induced to differentiate. Total RNA was harvested and relative EZH2 expression was analyzed by qRT-PCR and normalized to ribosomal protein L4 (RPL4) expression (2^−∆∆CT^). (Inset) Endogenous fold-change in the expression of EZH2 in satellite cells (control) during the differentiation program. Data represented are means ± SEM.

In our study, primary satellite cell cultures were allowed to progress through their myogenic lineage ex vivo for 48 h under proliferative conditions and another 48 h under differentiative conditions. EZH2 gene expression was analyzed every 24 h after plating for 96 h. After 48 h of proliferation, and just prior to induction to differentiate, EZH2 expression was reduced around 30% in satellite cells from the tributyrin treated piglets as compared to the control (*p* = 0.07) (Figure 1). EZH2 transcript expression between the treatments was similar 24 h after plating and 24 h after cells had been induced to differentiate, and by 48 h of differentiation, there were no treatment effects on EZH2 gene expression.

### 3.2. Genomic Landscape of H3K27me3 and H3K27ac

The results from our EZH2 gene expression analysis indicated an early reduction of EZH2 transcript production in satellite cells from animals fed tributyrin when compared to those not supplemented with tributyrin. These results were then compared with known targets of EZH2 methyltransferase activity and markers of satellite cell lineage. Satellite cells from tributyrin treated piglets (*n* = 3) and control piglets (*n* = 3) were analyzed for global levels of genome binding for H3K27me3 and H3K27ac histone marks by ChIP-Seq.

**Figure 2:**
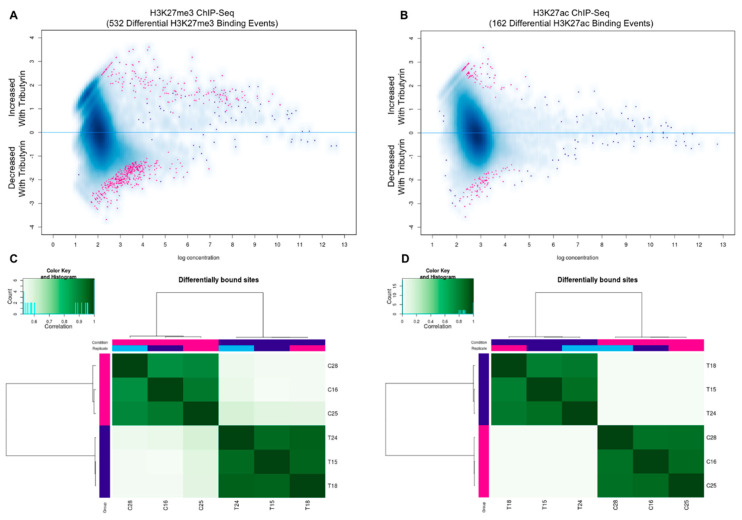
ChIP-Seq analysis of satellite cells selected randomly from control (C, *n* = 3) and tributyrin treated (T, *n* = 3) animals for H3K27me3 and H3K27ac histone marks. (Panels **A**,**B**) MA plot of tributyrin-control contrast. Significantly differentially bound sites shown in red. (Panels **C**,**D**) Unsupervised hierarchical clustering of differentially bound sites using H3K27me3 (**C**) and H3K27ac (**D**) profiles are shown with Pearson correlation. Grades are color coded with replicate numbers in the labels.

Tributyrin treatment resulted in 358 of the 532 differentially bound sites being significantly reduced for H3K37me3 enrichment based on edgeR analysis (FDR ≤ 0.05). Examination of H3K27ac revealed 162 differentially enriched regions with an even distribution of annotated regions between the treatment groups (FDR ≤ 0.10) (Figure 2). GO enrichment evaluation from differentially bound sites in satellite cells from tributyrin treated animals revealed significant decreases in the enrichment of repressive H3K27me3 marks associated with increased myoblast differentiation, negative regulators of cell proliferation, and microRNAs involved in satellite cell differentiation as compared to cells from the control animals. Conversely, sites involved with positive regulators of cell proliferation and increased stem cell maintenance were enriched for H3K27me3 in the tributyrin group (Table 1). We performed the same analysis with H3K27ac differentially bound peaks found in control and tributyrin clusters; tributyrin cluster genes were enriched for GO terms related to increase cell adhesion, TORC1 complex and microRNAs upregulated during myoblast differentiation. H3K27ac control cluster genes were enriched for GO terms related to increased ATP and calcium binding (Table 2).

### 3.3. Butyrate’s Impact on EZH2 Activity and Satellite Cell Myogenesis

Given the temporal reduction of EZH2 expression just prior to satellite cell differentiation and the reduction of the repressive H3K27me3 marks found near regions associated with the myogenic program in satellite cells from tributyrin fed animals, we sought to determine if the HDAC inhibitor butyrate may work through, or in conjunction with EZH2. To clarify whether these findings enhanced myogenic differentiation, we selectively silenced EZH2 transcript in satellite cells isolated from control animals. Cells were were exposed to either butyrate (0.5 mM, NaBu), RNAi targeting the EZH2 transcript (siEZH2), or both butyrate and siEZH2 (Mixed) during proliferation.

**Figure 3:**
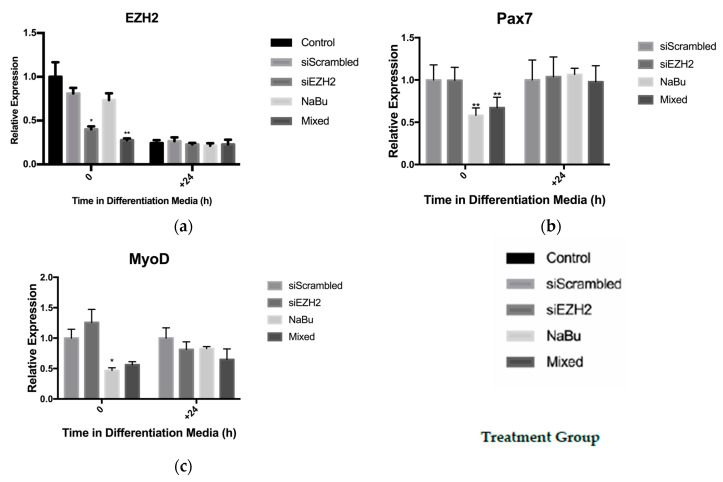
Effect of EZH2 knock-down and butyrate treatment on gene expression in satellite cells. Satellite cells from untreated control piglets were isolated and cultured under proliferative conditions until around 80% confluence, treated for 24 h, and then induced to differentiate. Satellite cell treatment groups: siRNA targeting the EZH2 transcript (siEZH2), a scrambled control siRNA (siScrambled), 0.5 mM sodium butyrate (NaBu), or both NaBu + siEZH2 (Mixed). Gene expression was analyzed by qRT-PCR and all genes of interest were normalized to RPL4. Relative expression of the genes of interest (**a**) EZH2, (**b**) Pax7, and (**c**) MyoD from the transfected treatments (siEZH2 and Mixed) were normalized to siScrambled control while the NaBu treatment was normalized to the untreated control (2^−∆∆CT^). Data represented are means ± SEM; * *p* < 0.05; ** *p* < 0.01.

**Figure 4:**
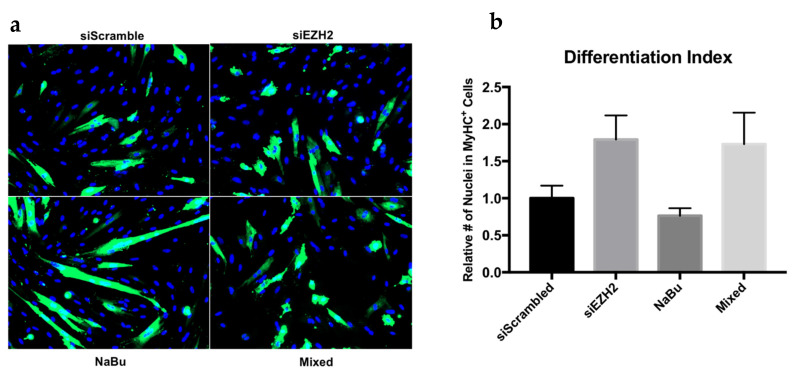
EZH2 knock-down and butyrate treatment effect on satellite cell differentiation as assessed by myosin heavy chain (MyHC) expression 72 h after differentiation. Satellite cells were cultured under proliferative conditions until around 80% confluence, treated for 24 h, and then induced to differentiate. Satellite cell treatment groups: siRNA targeting the EZH2 transcript (siEZH2), a scrambled control siRNA (siScrambled), 0.5 mM sodium butyrate (NaBu), or both NaBu + siEZH2 (Mixed). (**a**) Representative images of myotube formation revealed by immunostaining with anti-MyHC (green); nuclear counterstain with DAPI (blue). (**b**) The number of MyHC^+^ cells were counted and compared to total nuclei. The number of MyHC^+^ cells from the transfected treatments (siEZH2 and Mixed) were normalized to the siScrambled control while the NaBu treatment was normalized to the untreated control. Data represented are means ± SEM.

After 24 h of exposure to butyrate, satellite cell expression of the EZH2 transcript was not reduced as compared to the control. The siEZH2 treatment resulted in a >60% reduction (*p* < 0.01) in EZH2 transcript expression as compared to siScrambled control. An additive effect of the combination of butyrate and siEZH2 (Mixed) was seen with a 75% reduction (*p* < 0.01) in EZH2 gene expression compared to the siScrambled treatment (Figure 3A). After 24 h of differentiation (D+24), EZH2 expression was reduced as compared to levels during proliferation as has been previously reported [41], with no differences between treatments. Total RNA was also analyzed for the expression of the myogenic genes Pax7, MyoD, and myogenin. EZH2 knockdown did not have a significant effect on the expression of the myogenic genes after transfection or during differentiation. Treatment with NaBu alone and NaBu + siEZH2 did, however, result in reducing Pax7 (*p* < 0.01) and MyoD (*p* = 0.01) expression around 40% 24 h after treatment (Figure 3B,C), but did not affect myogenin expression (Figure not shown). After being induced to differentiate, no differences in myogenic gene expression were found between treatment groups. After 72 h of differentiation, satellite cells were fixed and stained for the contractile protein MyHC as an indicator of terminal differentiation. Satellite cells treated with siEZH2 had an approximately around 75% increase (*p* = 0.07) in MyHC^+^ cells relative to the siScrambled control, but EZH2 knockdown did not result in hypertrophied myotubes (Figure 4).

## 4. Discussion

Dietary inclusion of tributyrin resulted in a temporally hastened reduction in EZH2 expression in satellite cells ex vivo. Primary satellite cells from tributyrin treated piglets showed a reduction in EZH2 expression just prior to differentiation as compared to the control piglets. After 24 h in differentiation media, EZH2 expression was similar between the two groups. These findings indicate that those cells may start the differentiation program earlier or may be primed to initiate the differentiation machinery more quickly. There are published data that are consistent with this scenario, showing that an auto-regulatory loop exists in which the microRNA miR-214 (discussed below) targets the 3′UTR of EZH2 just prior to differentiation [56]. Given the reduction in EZH2 seen in satellite cells from treated animals, we performed ChIP-Seq to determine global genome enrichment of the H3K27me3 and H3K27ac histone marks.

The transcriptionally repressive H3K27me3 histone mark deposited by the Polycomb EZH2 methyltransferase protein has been implicated in regulating satellite cell specification and the differentiation process [6]. It has been demonstrated in other tissue specific stem cells that EZH2 expression is regulated by HDACs and can be altered with HDAC inhibitors in vitro [13]. Animals treated with butyrate have shown increased functionality in models of muscle pathology [5] and increased growth [57]; however, the molecular mechanisms leading to these outcomes has remained generally unaddressed. Our results suggest that dietary inclusion of butyrate is sufficient to alter the expression of EZH2 and the epigenetic landscape of satellite cells. Our data does not indicate that the reduction in EZH2 expression caused by butyrate is the sole mechanism through which butyrate may alter the myogenic program of satellite cells.

ChIP-Seq was performed on proliferating primary satellite cells and revealed that treatment with tributyrin resulted in a global reduction of H3K27me3 marks, but intriguingly did not result in the hyperacetylation of H3K27. It appears that other differentiation cues must be necessary in order to transition from what may be a poised state to an active state [39] despite HDAC inhibition. Our results showed that those regions with differential enrichment of H3K27me3 were associated with the GO terms related specifically to myoblast differentiation. Most importantly among these genes was the MRF MyoD which had a differentially enriched region 10kb upstream of the transcription start site that was reduced for H3K27me3 with tributyrin treatment. There is evidence that the homeoprotein Msx1, Msh homeobox-1, recruits the PRC2 complex to MyoD regulatory regions and results in increased repressive H3K27me3 being deposited in several regions [58]. Other genes that were associated with sites showing an overall reduction in H3K27me3 were related to the GO terms negative regulation of cell proliferation, cell cycle arrest, and plasma membrane adhesion molecules. While tributyrin treatment resulted in fewer H3k27me3 enriched regions as compared to the control, those regions that were enriched relative to the cells from the control treatment were associated with genes related to the positive regulation of cell proliferation. These results underscore the anti-proliferative and pro-differentiative properties attributed to butyrate [59] while also highlighting the epigenetic nature of these changes. Interestingly, our ChIP-Seq results indicate that tributyrin treatment resulted in differentially enriched regions around key miRNAs. Of note, miR-214 which regulates EZH2 expression [56], as detailed above, was enriched for the repressive H3K27me3 in the satellite cells of the control animals. This may explain one way in which EZH2 expression was reduced in the satellite cells from the treatment group. Also, H3K27me3 marks around miR-206 were reduced in satellite cells from the tributyrin treatment group. miR-206 has been implicated in repressing Pax7 and allowing for differentiation to take place [60]. Complementary to this, tributyrin treatment resulted in H3K27ac enrichment near both miR-181a and miR-210 which are strongly upregulated during satellite cell differentiation [61,62]. It appears that the beneficial effects of butyrate on satellite cells reported previously [10] may be due, at least in part, to a modulation of EZH2 activity.

To investigate whether butyrate’s primary mechanism of action occurs through the down regulation of EZH2 expression and subsequent hypomethylation of H3K27, we assessed the interaction of EZH2 depletion alone or in concert with butyrate exposure in proliferating satellite cells just prior to differentiation. We found that EZH2 knockdown alone had no effect on myogenic gene expression at any time point; however, butyrate significantly reduced Pax7 and MyoD gene expression during proliferation, both of which are required for proper satellite cell activation and proliferation [63,64]. Pax7 is required for satellite cell specification [21], but is also crucial for preventing precocious differentiation [25]. It is clear that butyrate must act through some other mechanism to silence Pax7, aiding in myogenic cell commitment. It has been determined that EZH2 is required for Pax7 gene silencing through progressive H3K27me3 deposition at the Pax7 promoter [7]. Immunostaining for MyHC^+^ satellite cells revealed that treatment with siEZH2 forced differentiation; however, it did not result in proper activation of the differentiation program as indicated by the failure of these myoblasts to form multinucleated myotubes. These findings are consistent with conditional ablation of SUZ12 (another component of PRC2) in myoblasts, with the exception that SUZ12 ablation resulted in enhanced myotube formation [8]. This may be in part due to the inability to assemble H3K27me3 marks about the Pax7 gene. In the absence of other differentiation cues, it was apparent that forcing the differentiation program too early had severe repercussions on late stage myogenesis as indicated by impaired myotube formation. The combined action of butyrate and siEZH2 was not enough to recover appropriate myotube formation. These results suggest that premature EZH2 ablation will inadvertently initiate the differentiation program without the appropriate mechanisms in place for satellite cell fusion or myotube hypertrophy.

## 5. Conclusions

**Figure 5:**
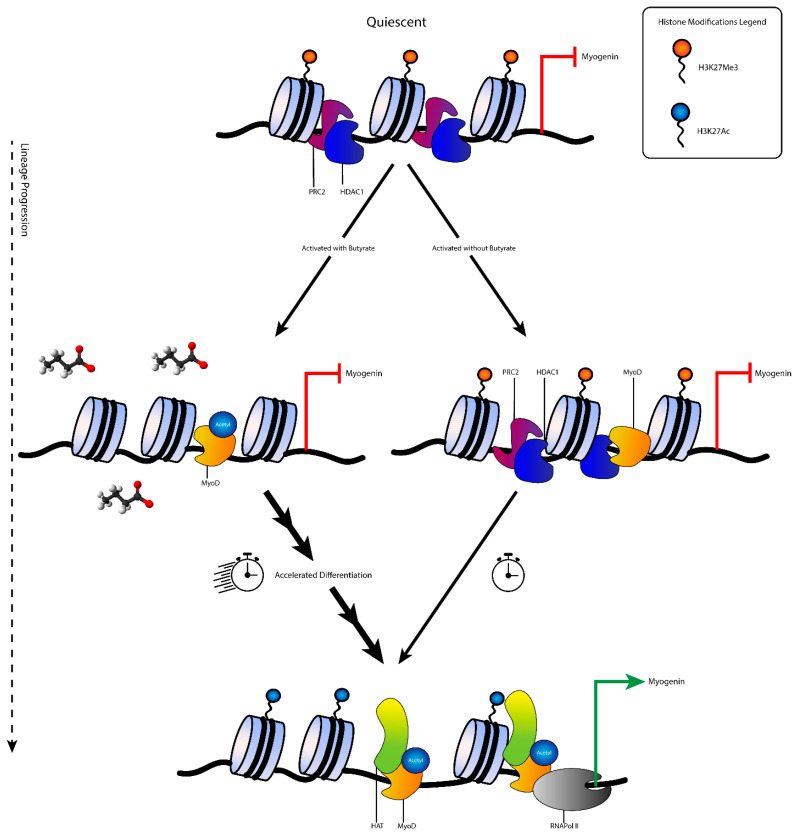
Proposed regulation of satellite cell activation and differentiation in the presence of butyrate. In undifferentiated, quiescent satellite cells lineage determination genes (i.e., myogenin) are not expressed due to the repressive H3K27me3 heterochromatin marks and lack of MyoD locating to the promoter region. Upon activation, MyoD is transcribed and locates to the promoter region of muscle genes (i.e., myogenin). Under normal conditions, MyoD is associated with HDAC1 and H3K27me3 still marks relevant muscle genes which prevent precocious differentiation. Satellite cells that are activated in the presence of butyrate allow the early acetylation of MyoD and removal of H3K27me3 marks. Butyrate inhibits HDAC1 and may interfere with PRC2 mediated trimethylation of H3K27. Inhibition of HDAC1 may also cause early PRC2 dislocation from muscle gene promoter regions. During differentiation cues, histone acetyl transferases (HATs) are recruited to muscle genes for deposition of the active H3K27ac euchromatin mark. Histone tail acetylation takes place during differentiation, but at a faster pace in the presence of butyrate while repressive PRC2 that has already disassociated from muscle gene promoters can now be recruited to the promoters of stemness genes.

We have demonstrated that dietary tributyrin supplementation had a genome-wide impact on H3K27me3 mark deposition in satellite cells. It appears that this reorganization of the genetic landscape is likely due to the altered activity of PRC2 methyltransferase enzyme EZH2 and the synergistic effect with the HDAC inhibitor butyrate. Our in vitro work showed that a reduction in EZH2 gene expression is not the only pro-myogenic differentiation cue that may be taking place when satellite cells are in the presence of butyrate. It has been suggested that an interaction exists where in which HDAC1 complexes with EZH2 and they function collectively to trimethylate H3K27 [6]. It is possible that the HDAC inhibitory effects may reduce the effect and recruitment of EZH2 to areas responsible for muscle gene expression, and that these marks are passed on to subsequent daughter cells (Figure 5). Further investigation is warranted into the hyperacetylation of MyoD, as well as the co-immunoprecipitation of the genome that is bound by both EZH2 and HDAC1. Taken together, these observations detail one way through which dietary supplementation of tributyrin may be accelerating the myogenic program.

## Figures and Tables

**Table 1:** Gene Ontology (GO) Analysis of H3K27me3 Differently Bound Peaks.

GO Categories Enriched in H3K27me3 from Satellite Cells Treated with Tributyrin				
GO Term	Associated Gene ID	Gene Name/Function	Fold Change	*p*-Value
Skeletal Muscle Cell/Myoblast Differentiation/Cell Differentiation	MYOD1	myogenic differentiation 1	−2.059	4.2 × 10^−4^
	SOX11	SRY-box 11	−2.653	3.0 × 10^−4^
	QKI	KH domain containing RNA binding	−1.398	4.7 × 10^−4^
	NTRK3	neurotrophic tyrosine kinase, receptor, type 3	−2.023	1.9 × 10^−4^
Negative Regulation of Cell Proliferation	DRD2	dopamine receptor D2	−2.508	3.1 × 10^−4^
	BTG4	BTG anti-proliferation factor 4	−2.002	1.3 × 10^−5^
	INSM1	INSM transcriptional repressor 1	−1.486	3.0 × 10^−4^
Negative Regulation of Cell Response to Growth Factor Stimulus	SLIT2	slit guidance ligand 2	−2.227	4.7 × 10^−4^
	CASK	calcium/calmodulin dependent serine protein kinase	−1.308	2.5 × 10^−4^
Cell Cycle Arrest	THBS1	thrombospondin 1	−1.753	4.9 × 10^−4^
	IL-12B	interleukin 12B	−2.251	1.8 × 10^−7^
Homophilic cell adhesion via plasma membrane adhesion molecules	CDH6	cadherin 6	−2.002	1.5 × 10^−5^
	CDH10	cadherin 10	−1.853	2.5 × 10^−4^
	FAT3	FAT atypical cadherin 3	−2.201	3.3 × 10^−4^
Micro-RNAs	MIR206	-----	−2.574	4.1 × 10^−4^
	MIR214	-----	−1.757	4.5 × 10^−4^
	MIR208B	-----	−1.525	5.9 × 10^−4^
	MIR130A	-----	2.862	2.0 × 10^−4^
Positive Regulation of Cell Proliferation	FGF17	fibroblast growth factor 17	2.496	1.2 × 10^−8^
	RASGRP4	RAS guanylyl releasing protein 4	1.743	2.9 × 10^−5^
Germ-line stem cell population maintenance	PIWIL2	piwi-like RNA-mediated gene silencing 2	2.502	1.1 × 10^−6^

Gene ontology (GO) enrichment analysis was performed with DAVID Bioinformatics Resources 6.8 to highlight the most relevant GO terms from the list of related gene IDs annotated by ChIPpeakAnno.

**Table 2:** Gene Ontology (GO) Analysis of H3K27ac Differently Bound Peaks.

GO Categories Enriched in H3K27ac from Satellite Cells Treated with Tributyrin				
GO Term	Associated Gene ID	Gene Name/Function	Fold Change	*p*-Value
Regulation of Cell Proliferation	DMNT1	DNA (cytosine-5-)-methyltransferase	−1.732	1.2 × 10^−4^
ATP Binding	MYO5A	myosin VA	−3.563	3.8 × 10^−5^
	PLK2	polo like kinase 2	−2.695	9.1 × 10^−7^
	EGFR	epidermal growth factor receptor	−2.643	1.3 × 10^−4^
Calcium Ion Binding	SCUBE3	signal peptide, CUB domain, EGF-like 3	−2.407	1.3 × 10^−4^
	ANXA10	annexin A10	−2.233	4.2 × 10^−5^
	ITPR3	inositol 1,4,5-triphosphate receptor 3	−2.022	2.7 × 10^−5^
Micro-RNAs	MIR128-1	-----	−2.360	1.4 × 10^−4^
	MIR181A-1	-----	1.964	3.8 × 10^−6^
	MIR210	-----	2.922	5.7 × 10^−7^
Cell Adhesion Via Plasma Membrane	CDH7	cadherin 7	2.819	5.6 × 10^−5^
	PCDH15	protocadherin 15	3.133	4.6 × 10^−7^
TORC1 Complex	RPTOR	regulatory associated protein of MTOR, complex 1	1.916	9.8 × 10^−7^

Gene ontology (GO) enrichment analysis was performed with DAVID Bioinformatics Resources 6.8 to highlight the most relevant GO terms from the list of related gene IDs annotated by ChIPpeakAnno.

## Data Availability

The ChIP-Seq datasets generated during and analyzed during the current study are available in the NCBI Short Read Archive repository, https://www.ncbi.nlm.nih.gov/bioproject/436619 (accessed on 5 December 2021). All other datasets generated during and/or analyzed during the current study are available from the corresponding author on reasonable request.

## Supplementary Materials

The following are available online at https://www.mdpi.com/article/10.3390/cells10123475/s1, Table S1: Primer and probe sequences used for gene expression analysis by quantitative RT-PCR.
