# Supplementary material for: Tributyrin, a Butyrate Pro-Drug, Primes Satellite Cells for Differentiation by Altering the Epigenetic Landscape
Source: Cells. 2021 Dec 9;10(12):3475. doi: 10.3390/cells10123475 (PMC8700657; doi:10.3390/cells10123475)
Supplement: Supplementary file 1 [file cells-10-03475-s001.zip › cells-1441061-supplementary.pdf]

cells MDPI

Table S1. Primer and probe sequences used for gene expression analysis by quantitative RT-PCR.

Gene Symbol	Gene ID	Primer Sequence 5′-3′	Probe and Sequence 5′-3′
PAX7[α]	100625823	F: CAGCAAGCCCAGACAGG<br>R: TCGGATCTCCCAGCTGAA	(HEX): TTGAGGAGTACAAGAGGGAGAACCCA
MYOD1[α]	407604	F: CCGACGGCATGATGGATTATAG<br>R: CGACACCGCAGCATTCTT	(FAM): AATAGGTGCCGTCGTAGCAGTTCC
MYOG[α]	497618	F: AGTGAATGCAGTTCCCACAG<br>R: AGGTGAGGGAGTGCAGATT	(Texas Red): CAACCCAGGGGATCATCTGCTC
RPL4[α]	100038029	F: TGGTGGTTGAAGATAAAGTTGAAAG<br>R: TGAGAGGCATAAACCTTCTTGAT	(Cy5): AACCAAGGAGGCTGTTCTGCTTCT
EZH2[β]	100625497	F: GCGGAAGCGTGTAAAATCAGA<br>R: CCTTCGCTGTTTCCACTCTT	----------
RPL4[β]	100038029	F: CAAGAGTAACTACAACCTTC<br>R: GAACTCTACGATGAATCTTC	----------

α- Multiplex, probe based qRT-PCR; β- Singleplex, SYBR Green qRT-PCR.
